# Myeloid cell-specific sirtuin 6 deficiency delays wound healing in mice by modulating inflammation and macrophage phenotypes

**DOI:** 10.1038/s12276-019-0248-9

**Published:** 2019-04-26

**Authors:** Jeung-Hyun Koo, Hyun-Young Jang, Youngyi Lee, Young Jae Moon, Eun Ju Bae, Seok-Kweon Yun, Byung-Hyun Park

**Affiliations:** 1 Department of Biochemistry and Molecular Biology, Chonbuk National University Medical School, Jeonju, Jeonbuk 54896 Republic of Korea; 2 College of Pharmacy, Woosuk University, Wanju, Jeonbuk 55338 Republic of Korea; 3 Department of Dermatology and Research Institute of Clinical Medicine, Chonbuk National University Medical School, Jeonju, Jeonbuk 54896 Republic of Korea; 4 Biomedical Research Institute, Chonbuk National University Hospital, Jeonju, Jeonbuk 54907 Republic of Korea

**Keywords:** Chronic inflammation, Chronic inflammation

## Abstract

We recently reported that myeloid cell-expressed sirtuin 6 (Sirt6) plays a crucial role in M1 macrophage polarization and chemotaxis. Given the prominent role of macrophages during wound repair and macrophage heterogeneity, we hypothesized that a Sirt6 deficiency in myeloid cells would delay skin wound closure by affecting the phenotypes of macrophages in wounds. To address this question, a full-thickness excisional lesion was made in the dorsal skin of myeloid cell-specific Sirt6 knockout (KO) and wild-type mice. Wound closure was delayed in the KO mice, which exhibited less collagen deposition, suppressed angiogenesis, and reduced expression of wound healing-related genes compared to the wild-type mice. Using immunohistochemical, flow cytometric, and gene-expression analyses of macrophage subpopulations from wound tissue, we identified increased infiltration of M1 macrophages with a concomitant decrease in M2 macrophage numbers in the KO mice compared to the wild-type mice. Consistent with the in vivo wound closure defects observed in the KO mice, keratinocytes and fibroblasts treated with KO macrophage-derived conditioned medium migrated slower than those treated with wild-type macrophage-derived conditioned medium. An analysis of downstream signaling pathways indicated that impaired Akt signaling underlies the decreased M2 phenotypic switching in KO mice. These results suggest that a macrophage phenotypic switch induced by Sirt6 deficiency contributes to impaired wound healing in mice.

## Wound healing: Protein deficiency hinders repair

The loss of a protein involved in regulating white cell populations at wound sites can delay healing. Macrophages, a type of white blood cell, are crucial to wound healing. The M1 type of macrophage triggers initial inflammation, consumes damaged tissues, and secretes other molecules that help healing. M1 cells switch to the M2 type during the later stages of wound repair. Seok-Kweon Yun and Byung-Hyun Park at Chonbuk National University Medical School, Jeonju, South Korea, and co-workers examined macrophage phenotypes in wounds, focusing on a protein involved in macrophage type called sirtuin 6 (Sirt6). They compared wound healing responses in wild type mice and those without myeloid Sirt6. In myeloid Sirt6-deficient mice, increased levels of M1 macrophages induced higher levels of inflammation. There was also failure in M2 phenotypic switching in myeloid Sirt6 KO mice, which further delayed wound healing.

## Introduction

Skin wound healing is a highly ordered process comprising several overlapping stages: (i) an inflammatory stage that cleans out debris and bacteria, (ii) a proliferative stage that refills the dermal wound space, and (iii) a long-term remodeling stage that involves the resolution of inflammation and reorganization of connective tissue into a scar^1^. Thus, the recruitment of inflammatory cells into the wound site is an initial event in the tissue repair process. Neutrophils form the first line of defense against infection and are a source of proinflammatory cytokines^2,3^. Macrophages also regulate wound healing by producing various growth factors such as transforming growth factor-β, basic fibroblast growth factor, and platelet-derived growth factor^4–6^. In response to these growth factors, epithelial cells proliferate and migrate to cover the wound, endothelial cells participate in angiogenesis, and fibroblasts contribute to the process of dermal healing^7^. In support of this view, suppressed recruitment of macrophages into wound sites impairs wound healing^8,9^. However, due to the release of proinflammatory and cytotoxic mediators, uncontrolled macrophage activity may also be detrimental to tissue repair^1^. Macrophages have been subdivided into two subpopulations based on their distinct gene-expression profiles: classically activated M1 macrophages and alternatively activated M2 macrophages^10^. Interestingly, not only does the number of macrophages infiltrating the wound site change but macrophage phenotypes also shift in the various stages of wound repair. In the inflammatory stage, M1 macrophages initiate an acute inflammatory response, whereas during the proliferative stage, M2 macrophages promote angiogenesis and granulation tissue formation^11^. These findings suggest that proper macrophage polarization is critical to effective wound healing. However, the mechanism that regulates macrophage polarization is unclear.

Sirtuins are a class of NAD-dependent histone deacetylases that consists of seven enzymes (Sirt1 to Sirt7) that differ in their cellular localization. Among the seven sirtuin family members, Sirt6 is localized in the nucleus and is involved in transcriptional silencing, genome stability, and longevity^12^. As a histone deacetylase, Sirt6 deacetylates histone H3 lysine 9 (H3K9)^13^ and histone H3 lysine 56 (H3K56)^14^ and represses the transcriptional activities of several transcription factors. As a nonhistone protein deacetylase, Sirt6 deacetylates forkhead box protein O1^15,16^, C-terminal binding protein interacting protein^17^, GCN5^18^, pyruvate kinase M2^19^, and GATA binding protein 3^20^. Since Sirt6 is a critical determinant of phenotypic switching and the migratory responses of macrophages^21^, we hypothesized that myeloid cell-expressed Sirt6 could play a role in the wound healing process. To test this hypothesis, we constructed myeloid cell-specific Sirt6 knockout (mS6KO) mice and studied the effects of Sirt6 deficiency on cell migration in vitro and inflammation and wound healing in vivo.

## Materials and methods

### Animals

*Sirt6*^*flox/flox*^ mice (B6;129-*Sirt6*^tm1Ygu^/J) and *LysM-Cre* mice (B6.129P2-*lyz2*^tm1(cre)Ifo^/J) were obtained from The Jackson Laboratory (Bar Harbor, ME, USA). *Sirt6*^*flox/flox*^ and homozygous *LysM-Cre* mice were crossed to obtain mS6KO mice. To avoid potential variations due to sex and/or genetic background, female mice from the F2 generation [*Sirt6*^*flox/flox*^; *LysM-Cre*^*+*^ (mS6KO) and *Sirt6*^*flox/flox*^; *LysM-Cre*^−^ (WT)] were used for all experiments. The mice had free access to food and water and were maintained in a room with controlled humidity (50%) and temperature (22 °C) on a 12-h light/dark cycle. All animal experiments were performed in accordance with the Guide for the Care and Use of Laboratory Animals published by the US National Institutes of Health (NIH Publication No. 85-23, revised 2011). The study protocol was approved by the Institutional Animal Care and Use Committee of Chonbuk National University (Permit No. CBNU 2015-083).

### Wound modeling

Twelve-week-old WT and KO mice were intraperitoneally anaesthetized with ketamine (100 mg/kg) and xylazine (5 mg/kg), shaved, and cleaned. An immediate-bonding adhesive (Grace Bio-Labs, Bend, OR, USA) was used to fix a splint to the skin. Full-thickness wounds were made in the doughnut region of the splint using 6-mm-diameter punches (Acuderm, Fort Lauderdale, FL, USA). Tegaderm film (3 M Health Care, St. Paul, MN, USA) was placed over the wounds to stabilize the wound site. The percentage of the initial wound that remained open was quantitated at different time points (days 0, 3, 5, 7, and 14). After the mice were sacrificed, the skin wounds were collected for histopathological analysis and RNA isolation.

### Histology

Tissue was removed and immediately placed in fixative (10% formalin solution in 0.1 M phosphate-buffered saline (PBS)). Histological sections (5 μm) were cut from formalin-fixed, paraffin-embedded tissue blocks. To compare histopathology between lesions, we harvested skin near the center of the wound. The tissue sections were stained with hematoxylin-eosin (H&E) under standard conditions. Immunohistochemical staining was performed using a DAKO Envision system (DAKO, Carpinteria, CA, USA), which uses dextran polymers conjugated with horseradish peroxidase to avoid contamination with endogenous biotin. After deparaffinization, the tissue sections were treated using a microwave antigen-retrieval procedure with 0.01 M sodium citrate buffer. After blocking endogenous peroxidase activity, the sections were incubated with Protein Block Serum-Free (DAKO) to block nonspecific staining. The sections were then incubated with antibodies against F4/80 and von Willebrand factor (vWF; Millipore, Beverly, MA, USA). Peroxidase activity was detected with 3-amino-9-ethyl carbazole. The number of F4/80-positive macrophages was counted in five microscopic fields (magnification, 100×) for each sample in the fields with the highest numbers of F4/80-positive macrophages. The results are expressed as the average number of F4/80-positive macrophages per field. Masson’s trichrome staining was performed with a commercial kit from Abcam (ab150686, Cambridge, UK). Double-staining immunofluorescence analysis was performed to determine the types of macrophages in the skin wounds. Frozen sections were incubated with a combination of anti-F4/80 and anti-iNOS antibodies (Santa Cruz Biochemicals, Dallas, TX, USA) or anti-F4/80 and anti-MRC1 antibodies (also known as CD206; Abcam) at 4 °C overnight. Myofibroblasts were identified by staining with antibodies to vimentin (Santa Cruz Biochemicals) and α-smooth muscle actin (α-SMA; Abcam). After incubation with the corresponding fluorochrome-conjugated secondary antibodies, the sections were mounted and visualized using a LSM510 confocal laser scanning microscope (Carl Zeiss, Oberkochen, Germany).

### ELISA analysis

Tissue levels of TNF-α, IL-1β, IL-6, IL-4, IL-13, and IL-10 were measured using specific ELISA kits (all from eBioscience, San Diego, CA, USA).

### Flow cytometric analysis

Wound cells were isolated by an enzymatic digestion with collagenase V. For flow cytometric analysis, Fc receptors were blocked with mouse SeroBlock FcR (CD16/CD32, eBioscience). The cells were stained with a PerCP- or FITC-conjugated anti-F4/80, a FITC- or PE-conjugated anti-Ly6g, an APC-conjugated anti-CD11b, a PE-conjugated anti-Ly6c, or a FITC-conjugated anti-MHCII antibody for 30 min at 4 °C. After washing with FACS buffer (2% fetal bovine serum (FBS) in PBS) three times, the cells were analyzed using an Accuri flow cytometer (BD Biosciences, San Jose, CA, USA).

### Cell culture

The human keratinocyte cell line HaCaT was kindly donated by Dr. Jeong HS (Chonnam National University Medical School, Gwangju, Korea). The cells were grown in Dulbecco’s modified Eagle’s medium (Lonza, Walkersville, MD, USA) supplemented with 10% (v/v) FBS and antibiotics (100 U/ml penicillin and 100 mg/ml streptomycin) at 37 °C in a humidified atmosphere with 5% CO_2_. A mouse dermal fibroblast cell line (MDFB) was obtained from iXCells Biotechnologies (San Diego, CA, USA) and grown in fibroblast growth medium (iXCells Biotechnologies). Adenoviruses expressing *Sirt6* (AdSirt6) and β-galactosidase (AdLacZ) were prepared as described previously^22^.

### Preparation of conditioned medium and the wound scratch assay

Bone marrow was isolated from the femurs and tibias of WT and KO mice and cultured in α-MEM (Invitrogen, Carlsbad, CA, USA) supplemented with 10% FBS. Floating cells were defined as bone marrow macrophages (BMMs). To prepare conditioned medium (CM), BMMs (2 × 10^6^) were grown in α-MEM supplemented with 10% FBS. Confluent cells were treated with TNF-α (10 ng/ml), IL-1β (10 ng/ml), and IL-6 (10 ng/ml) for 3 h; washed 3 times; and cultured for a further 24 h; then, the supernatants were collected and used. Cell migration was assessed by determining the ability of the cells to move into a cell-free area in a two-dimensional scratch assay. Briefly, HaCaT cells (2 × 10^6^ cells) or MDFB cells (2 × 10^6^ cells) were grown in a 12-well plate. When cell confluence reached 90% or higher, fresh medium containing 10 μg/ml mitomycin C was added for 2 h. The cells in the center of the well were scratched with a 100-μl sterile pipette tip to create a cell-free area. The medium was changed to WT or KO BMM-derived CM. The scratched area was photographed using a microscopy system (Carl Zeiss) soon after scratching and 12 and 36 h later. The scratch area was measured using iSolution DT 36 software (Carl Zeiss).

### M2 polarization

BMMs grown in α-MEM were stimulated with IL-4 (10 ng/ml, Invitrogen) and macrophage colony-stimulating factor (10 ng/ml, Thermo Fisher Scientific, Waltham, MA, USA) for 6 h. To exogenously express Akt in BMMs, cells were transduced with adenoviruses expressing a constitutively active form of Akt (S473D/T308D, AdAkt). The adenoviruses were a kind gift from Dr. Ahn J.Y. (Sungkyunkwan University, Suwon, Korea)^23^.

### Western blotting

Cells were homogenized in Mammalian Protein Extraction Reagent (Thermo Fisher Scientific). The homogenates (20 μg of total protein) were separated by sodium dodecyl sulphate polyacrylamide gel electrophoresis and transferred to nitrocellulose membranes. The blots were probed with primary antibodies against Sirt6 (Abcam), p-Akt, Akt, p-FoxO1, p-STAT6 (Cell Signaling Technology, Beverly, MA, USA), HSP90, α-tubulin, GAPDH (Bioworld, Irving, TX, USA), Arg1 (Santa Cruz Biochemicals), and Ym1 (STEMCELL Technologies, Vancouver, Canada). Immunoreactive bands were detected with a Las-4000 imager (GE Healthcare Life Science, Pittsburgh, PA, USA).

### RNA isolation and real-time RT-PCR

Total RNA was extracted from tissue using Trizol reagent (Invitrogen). RNA was precipitated with isopropanol and dissolved in diethyl pyrocarbonate-treated distilled water. First-strand cDNA was generated with oligo dT-adaptor primers by reverse transcription (TaKaRa). Specific primers were designed using qPrimerDepot (http://mouseprimerdepot.nci.nih.gov, Table S1). The real-time reverse transcription polymerase chain reaction (RT-PCR) reaction systems had a final volume of 10 μl and contained 10 ng of reverse-transcribed total RNA, 200 nM forward and reverse primers, and a PCR master mix. RT-PCR was performed in 384-well plates using the ABI Prism 7900HT Sequence Detection System (Applied Biosystems, Foster City, CA, USA). Reverse transcription and PCR were performed using a One-Step RT-PCR kit (Invitrogen). The PCR products were separated by electrophoresis on 2% agarose gels, followed by staining with ethidium bromide.

### Statistical analyses

Data are expressed as the mean ± standard error of the mean. GraphPad Prism software was used to perform the statistical analyses (GraphPad Prism version 5.2, San Diego, CA, USA). Significant differences between groups were determined using an unpaired Student’s *t* test. A *p* value less than 0.05 was considered significant.

## Results

### Wound healing is impaired in mS6KO mice

**Fig. 1:**
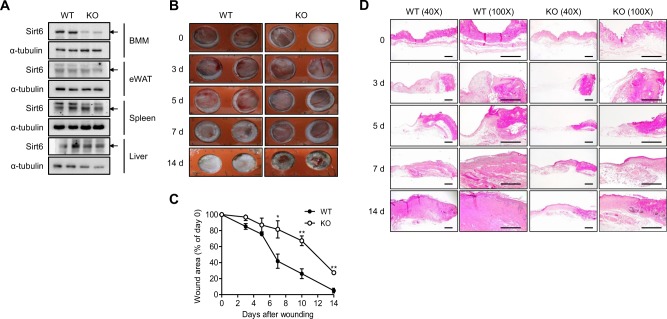
Delayed wound healing in mS6KO mice. **a** The process used to generate myeloid cell-specific Sirt6 knockout (mS6KO) mice is shown. **b** Excisional wounds were made by 6-mm-diameter punches. The wound sites were photographed at the indicated time. A day 0 picture was taken immediately after injury. Representative results from five individual mice from each group are shown. **c** Wound photographs were analyzed at the indicated times to determine wound closure in WT and KO mice. Values represent the mean ± SEM (*n* = 8). ^*^p < 0.05 and ^**^p < 0.01 vs. WT. **d** Histological sections of mouse skin wounds were examined by H&E staining. Bars = 250 μm

**Fig. 2:**
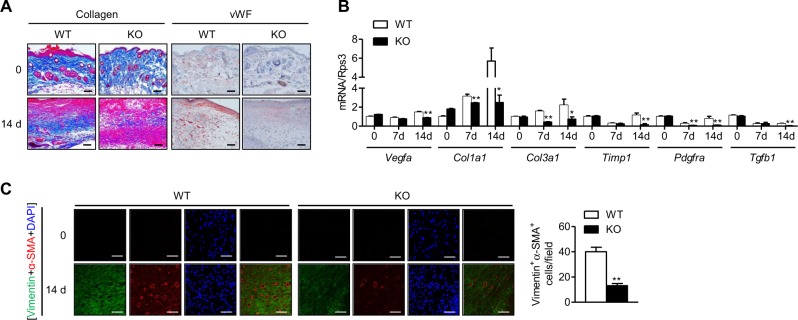
Decreases in collagen deposition and angiogenesis in mS6KO mice. **a** Representative photomicrographs of wounds stained with Masson’s trichrome or immunostained with an anti-vWF antibody at 0 and 14 days after injury are shown (original magnification, 200×). Bars = 50 μm. **b** The mRNA levels of extracellular matrix-related genes in wounds were determined by real-time RT-PCR (*n* = 8). **c** Skin wounds of WT and KO mice were double stained for vimentin and α-SMA expression to identify myofibroblasts. The vimentin and α-SMA-positive cells were counted in sections of the wounds harvested on day 14 (*n* = 6). Values represent the mean ± SEM. ^*^p < 0.05 and ^**^p < 0.01 vs. WT

To understand the role of myeloid cell-expressed Sirt6 in excisional wound healing, we generated mS6KO mice by breeding *Sirt6*^*flox/flox*^ mice with *LysM-Cre* mice. By Western blot analysis, the successful deletion of Sirt6 was confirmed in BMMs from mS6KO mice (Fig. 1a). Excision wounds were made in the dorsal skin of female mS6KO mice and their WT littermates. We observed slower wound healing in the mS6KO mice than in the WT mice, suggesting that the myeloid cell-specific Sirt6 deficiency delayed wound healing (Fig. 1b, c). Histological comparison of the wounds further confirmed delayed wound closure in the mS6KO mice (Fig. 1d). Trichrome staining of tissue harvested on day 14 after wounding showed reduced collagen content in the granulation tissue of the mS6KO mice (Fig. 2a). Similarly, the intensity of vWF-positive immunostaining was decreased in KO tissue, indicating that angiogenesis was also suppressed in the mS6KO mice (Fig. 2a). The mRNA levels of extracellular matrix genes (*Col1a1*, *Col3a1*, *Timp1, Pdgfra*, and *Tgfb1*) and the angiogenesis gene *Vegfa* were significantly suppressed in the skin tissue of the mS6KO mice (Fig. 2b). Consistent with these results, the number of myofibroblasts (vimentin^+^α-SMA^+^ cells) was significantly decreased in the skin wounds of the mS6KO mice (Fig. 2c). Collectively, these data suggest that myeloid cell-specific Sirt6 deficiency delays wound healing through the repression of collagen deposition, epithelial regrowth, and angiogenesis.

### Myeloid cell-specific Sirt6 deficiency decreases M2 macrophage infiltration into skin wounds

**Fig. 3:**
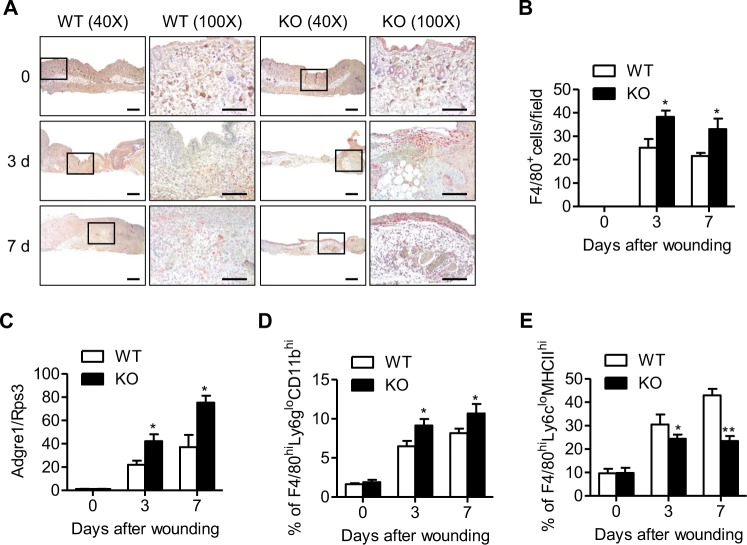
Increase in macrophage infiltration into wound sites in mS6KO mice. **a** Immunohistochemistry was used to identify infiltrating macrophages (F4/80^+^ cells) at 0, 3, and 7 days after injury. Bars = 50 μm. **b** F4/80-positive macrophages were counted in wound sections. **c** The mRNA level of F4/80 (*Adgre1*) in the wound site was determined by real-time RT-PCR (*n* = 8). **d**, **e** Single-cell suspensions were prepared by enzymatically digesting and gently dissociating skin wounds. After excluding dead cells, the cells were analyzed by flow cytometry to identify all macrophages (F4/80^hi^Ly6g^lo^CD11b^hi^) and M2 macrophages (F4/80^hi^Ly6c^lo^MHCII^hi^) (*n* = 5–6). Values represent the mean ± SEM. ^*^p < 0.05 and ^**^p < 0.01 vs. WT

We examined the infiltration of macrophages, which are critical inflammatory cells for wound healing, into wound sites by counting F4/80-positive cells. The accumulation of F4/80-immunopositive cells was significantly increased in mS6KO mice compared to WT mice (Fig. 3a, b). Real-time RT-PCR for F4/80 mRNA (*Adgre1*) expression and flow cytometric analysis for wound-associated macrophages (F480^hi^Ly6g^lo^CD11b^hi^) corroborated the increased accumulation of macrophages in mS6KO mice compared to WT mice (Figs. 3c, d, and S1A).

**Fig. 4:**
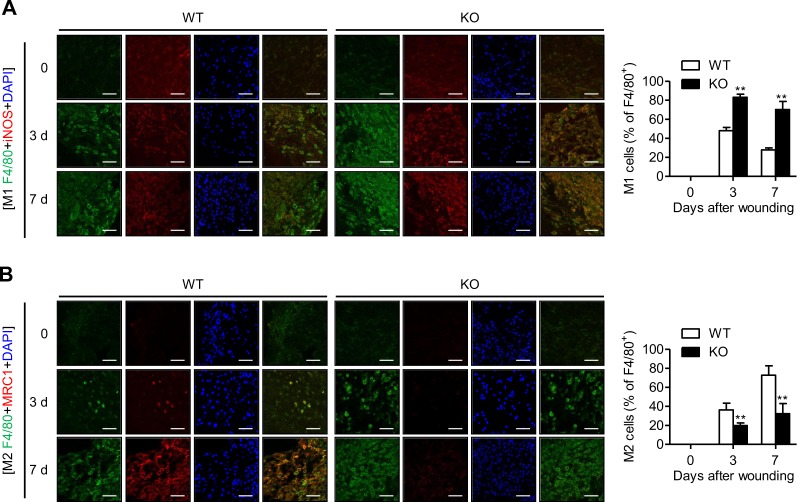
Decrease in the number of M2 macrophages in wound sites in mS6KO mice. The skin wounds of WT and KO mice were double stained for F4/80 and iNOS to identify M1 macrophages (**a**) or F4/80 and MRC1 to identify M2 macrophages (**b**). Digitally merged signals are shown in the right panels. Bars = 50 μm. Representative results from three independent experiments are shown

**Fig. 5:**
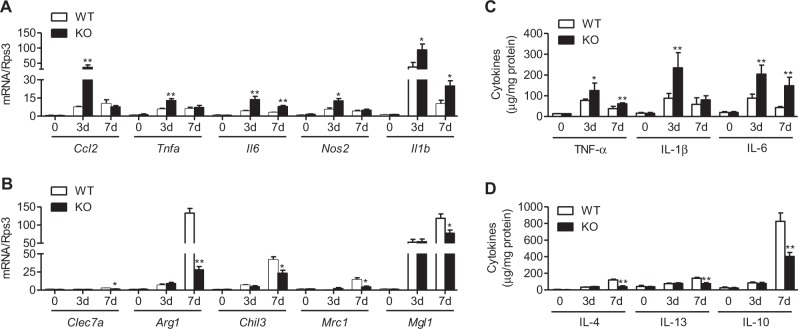
Temporal changes in the expression of markers for M1 and M2 macrophages in wound sites. **a**, **b** The mRNA levels of M1 and M2 marker genes in the wound site were determined by real-time RT-PCR (*n* = 8). **c**, **d** The levels of M1 and M2 cytokines in the tissue were measured using ELISA (*n* = 6). Values represent the mean ± SEM. ^*^p < 0.05 and ^**^p < 0.01 vs. WT

Next, we immunostained skin wounds with an M1-specific anti-iNOS antibody and M2-specific anti-MRC1 antibody. Myeloid cell-specific Sirt6 deficiency increased the number of F4/80^+^iNOS^+^ macrophages and decreased the number of F4/80^+^MRC1^+^ macrophages in the skin wounds of mS6KO mice compared to those of WT mice (Fig. 4a, b). Flow cytometric analysis also showed a decrease in the accumulation of M2 macrophages (F480^hi^Ly6c^lo^MHCII^hi^) in the skin wounds of mS6KO mice (Figs. 3e and S1B). Consistently, mRNA expression levels for M1 marker genes (*Ccl2*, *Tnfa*, *Il6*, *Il1b*, *Nos2*, and *Il1b*) and M1 cytokines (TNF-α, IL-1β, and IL-6) were increased, while the levels of M2 marker genes (*Clec7a*, *Arg1*, *Chil3*, *Mrc1*, and *Mgl1*) and M2 cytokines (IL-4, IL-13, and IL-10) were decreased in mS6KO tissue (Fig. 5a–d). These results indicate a decrease in the accumulation of M2-type macrophages in the skin wounds of mS6KO mice.

### CM from mS6KO BMMs decreases scratch wound closure

**Fig. 6:**
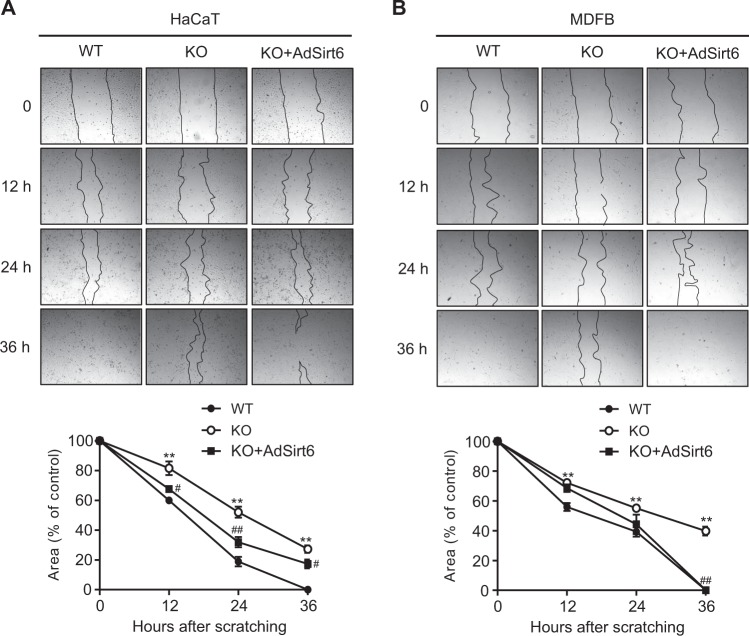
Delayed migration of skin keratinocytes and fibroblasts in wound scratch assays. Bone marrow macrophages (BMMs, 2 × 10^6^) isolated from WT or KO mice were treated with TNF-α (10 ng/ml), IL-1β (10 ng/ml), and IL-6 (10 ng/ml) for 3 h, and then conditioned medium (CM) was harvested. Scratch wounds were made in monolayers of HaCaT (**a**) and MDFB cells (**b**). For rescue experiments, KO BMMs were transduced with AdSirt6. Representative images show the progression of wound closure at the indicated times. The scratch area is indicated by a dotted line. The rate of wound closure was calculated by measuring the scratch area under the same magnification at different times. Values represent the mean ± SEM (*n* = 8) ^**^p < 0.01 vs. WT; ^#^*p* < 0.05; and ^##^*p* < 0.01 vs. KO

To examine whether secreted factors from M1 macrophages suppress cell migration during wound closure, we conducted scratch wound assays (Fig. S2). We chose epidermal keratinocytes (HaCaT cells) and dermal fibroblasts (MDFB cells) for the cell migration study. CM from KO BMMs significantly decreased the rate of HaCaT cell migration compared to CM from WT BMMs (Fig. 6a). Complete gap closure was seen with treatment with CM from WT BMMs by 36 h, as opposed to treatment with CM from KO BMMs resulting in only 58% closure at the same time. In experiments with MDFB cells, the overall results were similar: the fibroblasts treated with CM from WT BMMs migrated more quickly to fill the scratch area than those treated with CM from KO BMMs (Fig. 6b). Next, we assessed whether exogenous Sirt6 expression in KO BMMs could rescue the migratory defect seen in HaCaT and MDFB cells. Ectopic overexpression of Sirt6 in KO BMMs rescued cells from the migratory defect (Fig. 6a, b).

### Myeloid cell-specific Sirt6 deficiency suppresses M2 polarization through downregulation of the PI3K-Akt pathway

**Fig. 7:**
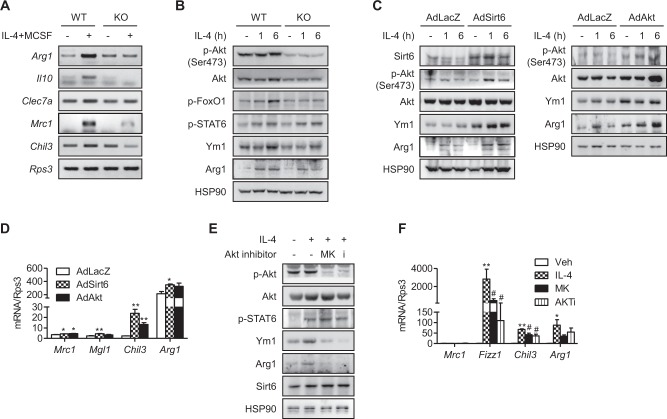
Regulation of M2 polarization by Sirt6. **a** BMMs were treated with 10 ng/ml IL-4 and 10 ng/ml MCSF for 6 h, and the expression patterns of M2 marker genes were compared by RT-PCR. **b** BMMs were treated with 10 ng/ml IL-4 for the indicated periods, and then whole-cell extracts were generated and analyzed by Western blotting. **c**, **d** BMMs from mS6KO mice were transduced with an adenovirus expressing β-galactosidase (AdLacZ), Sirt6 (AdSirt6), or CA-Akt (AdAkt) at a multiplicity of infection (MOI) of 100 and treated with IL-4 for the indicated times. Protein and mRNA levels were analyzed. **e**, **f** BMMs from WT mice were treated with 10 ng/ml IL-4 and 10 ng/ml MCSF for 6 h with or without Akt inhibitors (8 μM MK2206 and 10 μM Akti), and then the protein levels in the whole cell extract and mRNA levels of M2 marker genes were determined. Values represent the mean ± SEM (*n* = 3). ^*^p < 0.05 and ^**^p < 0.01 vs. AdLacZ or Veh; ^#^*p* < 0.05 vs. IL-4. Veh Vehicle, MK MK2206, i Akti

To explain the decreased number of M2 macrophages in skin wounds in mS6KO mice, we compared M2 polarization in vitro. BMMs isolated from mS6KO mice expressed markedly lower levels of M2 marker genes (*Arg1*, *Il10*, *Clec7a*, *Mrc1*, and *Chil3*) in IL-4-stimulated M2-polarizing conditions than BMMs from WT mice (Fig. 7a). The classic IL-4 signaling pathway involves the activation of phosphoinositide 3-kinase (PI3K)-Akt through the recruitment of IRS-1/2^24^. We observed increased levels of p-Akt and the downstream molecule p-FoxO1 after IL-4 stimulation in BMMs from WT mice, whereas this increase was not observed in BMMs from mS6KO mice (Figs. 7b and S3A). In addition to activating Akt, IL-4 treatment also leads to the activation of JAK2-STAT6^25^. The phosphorylation levels of STAT6 were similar in BMMs from mS6KO and WT mice (Fig. 7b), indicating that the change in M2 marker expression was mediated through the downregulation of components of the PI3K-Akt pathway and not through the JAK2-STAT6 pathway. To further investigate the regulation of the PI3K-Akt signaling pathway by Sirt6 under M2-polarizing conditions, we transduced BMMs with either Sirt6 (AdSirt6) or a constitutively active form of Akt (AdAkt). Treatment with IL-4 led to a moderate increase in M2 marker protein expression in the BMMs from mS6KO mice expressing Sirt6 or Akt (Figs. 7c and S3B). Accordingly, the levels of M2 marker genes were increased in the Sirt6- or Akt-overexpressing KO cells (Fig. 7d). The role of Akt signaling in the enhancement of M2 polarization was further tested by blocking Akt signals with MK2206 or Akti. When WT BMMs were pretreated with these Akt inhibitors prior to stimulation with IL-4, Akt phosphorylation and M2 marker gene expression were markedly suppressed (Figs. 7e, S3C, and 7F). Finally, consistent with these in vitro results, in vivo results showed that Akt phosphorylation was increased in skin wounds in WT mice but was significantly impaired in skin wounds in KO mice (Fig. S4).

## Discussion

In this study, we demonstrated that myeloid cell-specific Sirt6 deficiency leads to delayed wound closure compared to WT control. This aberrant wound closure pattern was associated with the augmented infiltration of macrophages that failed to phenotypically switch from M1 to M2 macrophages.

Previously, Thandavarayan et al.^26^ observed that Sirt6 knockdown impairs diabetic wound closure with concomitantly increased levels of oxidative stress, inflammatory cytokines, and NF-κB activation in skin wounds. Similarly, the sirtuin activator resveratrol accelerates wound repair by increasing keratinocyte proliferation, while the sirtuin inhibitor sirtinol retards wound closure^27^. More recently, Hu et al.^28^ showed that Sirt6 KO mice display delayed and incomplete healing of the cornea after wounding. These reports suggest a beneficial role for sirtuin (specifically Sirt6) activation in wound healing. Based on this background, we aimed to understand how a myeloid cell-specific Sirt6 deficiency would affect macrophage infiltration and wound closure using mS6KO mice. Our results showed that the number of macrophages in excisional wounds was markedly increased in mS6KO mice. The significantly increased expression of a macrophage chemoattractant, CCL2, in mS6KO mice is possibly responsible for the increased infiltration of macrophages seen in the wounds of this strain compared to those of WT mice. Remarkably, we clearly demonstrated heterogeneity in the macrophage populations recruited to the wounds of mS6KO mice. The expression levels of established M1-specific marker genes were upregulated in skin wounds in mS6KO mice, while the levels of M2 marker genes were downregulated. These results imply that the wound environment in mS6KO mice favors the proinflammatory M1 status.

Ample evidence points to a phenotypic switch from an M1 to an M2 macrophage in the process of wound repair^29^. In the early inflammatory phase, M1 macrophages are the predominant cells in the tissue and are involved in the clearance of pathogens and dead cells and in the modulation of the adaptive immune system. In the later proliferative phase, M2 macrophages contribute to the resolution of inflammation and tissue remodeling. While STAT6 has been shown to be required for IL-4 to exert its effects^24^, the level of p-STAT6 was shown to be unaffected by Sirt6 deficiency, indicating that IL-4 impacts other signaling pathways. We observed enhanced levels of p-Akt in BMMs after exposure to IL-4, a finding consistent with that of a previous study showing that the activation of the PI3K-Akt pathway results in stronger polarization of macrophages toward the M2 phenotype^25^. Moreover, the transduction of KO BMMs with CA-Akt selectively restored the expression of M2 marker genes. These results indicate that the activation of the PI3K-Akt pathway is required for the expression of M2 markers; however, future studies are needed to determine how Sirt6 deficiency affects the PI3K-Akt pathway.

Using in vivo and in vitro approaches, we demonstrated that myeloid cell-specific Sirt6 deficiency utilizes the following mechanisms to delay wound closure. First, more proinflammatory cytokines such as TNF-α, IL-1β, and IL-6 were produced in mS6KO mice. It is well known that M1-type macrophages are a source of proinflammatory cytokines. These cytokines alter the normal functioning of epithelial cells and dermal fibroblasts and ultimately delay the rates of re-epithelialization (epithelium closure) and wound closure (dermis closure)^29^. Second, we observed a lower number of vWF-positive cells in mS6KO mice than in WT mice. M1 macrophages are described as having an antiangiogenic profile, which is considered detrimental to recovery after an inflammatory event^30,31^. Third, the delayed wound closure observed in mS6KO mice was accompanied by decreases in the migration of keratinocytes and dermal fibroblasts, suggesting that myeloid cell-specific Sirt6 deficiency might impair epithelial and dermal closure by suppressing cell migration. Although the underlying mechanisms by which myeloid cell-specific Sirt6 deficiency suppresses keratinocyte and dermal fibroblast migration remain elusive, these findings suggest, for the first time, that myeloid cell-specific Sirt6 deficiency has a deleterious role in excisional wound healing. Therefore, myeloid cell-specific Sirt6 activation might be a therapeutic strategy for accelerating wound healing.

## Supplementary information

Supplementary Materials
