# Supplementary material for: Myeloid cell-specific sirtuin 6 deficiency delays wound healing in mice by modulating inflammation and macrophage phenotypes
Source: Exp Mol Med. 2019 Apr 26;51(4):48. doi: 10.1038/s12276-019-0248-9 (PMC6486573; doi:10.1038/s12276-019-0248-9)
Supplement: Supplementary file 1 — Supplementary Materials [file 12276_2019_248_MOESM1_ESM.pdf]

Myeloid sirtuin 6 deficiency delays wound healing in mice by modulating inflammation and macrophage phenotype

Jeung-Hyun Koo[1*], Hyun-Young Jang[1*], Youngyi Lee[1*], Young Jae Moon[1], Eun Ju Bae[2], Seok-Kweon Yun[3,4¶], and Byung-Hyun Park[1¶]

[1]Department of Biochemistry and Molecular Biology, Chonbuk National University Medical School, Jeonju, Jeonbuk 54896, Republic of Korea

[2]College of Pharmacy, Woosuk University, Wanju, Jeonbuk 55338, Republic of Korea

[3]Department of Dermatology and Research Institute of Clinical Medicine, Chonbuk National University Medical School, Jeonju, Jeonbuk 54896, Republic of Korea

[4]Biomedical Research Institute, Chonbuk National University Hospital, Jeonju, Jeonbuk 54907, Republic of Korea

Contents

1. Supplementary Table
2. Supplementary Figures

1. Supplementary Table

Table S1. Sequences and accession numbers for primers (forward, FOR; reverse, REV) used in real-time RT-PCR and PCR analyses

Gene	Sequences for primers	Accession No.
Adgre1	FOR: TTTCCTCGCCTGCTTCTTC	NM_010130
	REV: CCCCGTCTCTGTATTCAACC	
Tnfa	FOR: AGGGTCTGGGCCATAGAACT	NM_013693
	REV: CCACCACGCTCTTCTGTCTAC	
Il1b	FOR: GGTCAAAGGTTTGGAAGCAG	NM_008361
	REV: TGTGAAATGCCACCTTTTGA	
Il6	FOR: ACCAGAGGAAATTTTCAATAGGC	NM_031168
	REV: TGATGCACTTGCAGAAAACA	
Ccl2	FOR: ATTGGGATCATCTTGCTGGT	NM_011333
	REV: CCTGCTGTTCACAGTTGCC	
Mgl1	FOR: ATGATGTCTGCCAGAGAACC	NM_010796
	REV: ATCACAGATTTCAGCAACCTTA	
Nos2	FOR: TTCTGTGCTGTCCCAGTGAG	NM_010927
	REV: TGAAGAAAACCCCTTGTGCT	
Arg1	FOR: CAGAAGAATGGAAGAGTCAG	NM_007482
	REV: CAGATATGCAGGGAGTCACC	
Clec7a	FOR: GTGCAGTAAGCTTTCCTGGG	NM_020008
	REV: TCCCGCAATCAGAGTGAAG	
Il10	FOR: TGTCAAATTCATTCATGGCCT	NM_020008
	REV: ATCGATTTCTCCCCTGTGAA	
Mrc1	FOR: CAGGTGTGGGCTCAGGTAGT	NM_010548
	REV: TGGCATGTCCTGGAATGAT	
Chil3	FOR: GGGCATACCTTTATCCTGAG	NM_009892
	REV: CCACTGAAGTCATCCATGTC	
Col1a1	FOR: TAGGCCATTGTGTATGCAGC	NM_007742
	REV: ACATGTTCAGCTTTGTGGACC	
Col3a1	FOR: AAGGCGAATTCAAGGCTGAA	NM_009930
	REV: TGTGTTTAGTACAGCCATCCTCTA	
Timp1	FOR: AGGTGGTCTCGTTGATTCGT	NM_011593
	REV: GTAAGGCCTGTAGCTGTGCC	
Pdgfra	FOR: CGCCTGCAAGTGTGAGACAG	NM_011058
	REV: CGCCTGCAAGTGTGAGACAG	
Tgfb1	FOR: AAGACACATTTGGCCCTGAC	NM_011577
	REV: CCACAGAACTTAGCCCGGTA	
Vegfa	FOR: AGAGCA ACATCACCA GCAG	NM_009505
	REV: CAGTGAACGCTCCAGGATTT	
Rps3	FOR: AATGAACCGAAGCACACCATAG	NM_012052
	REV: ATCAGAGAGTTGACCGCAGTTG	

2. Supplementary Figures

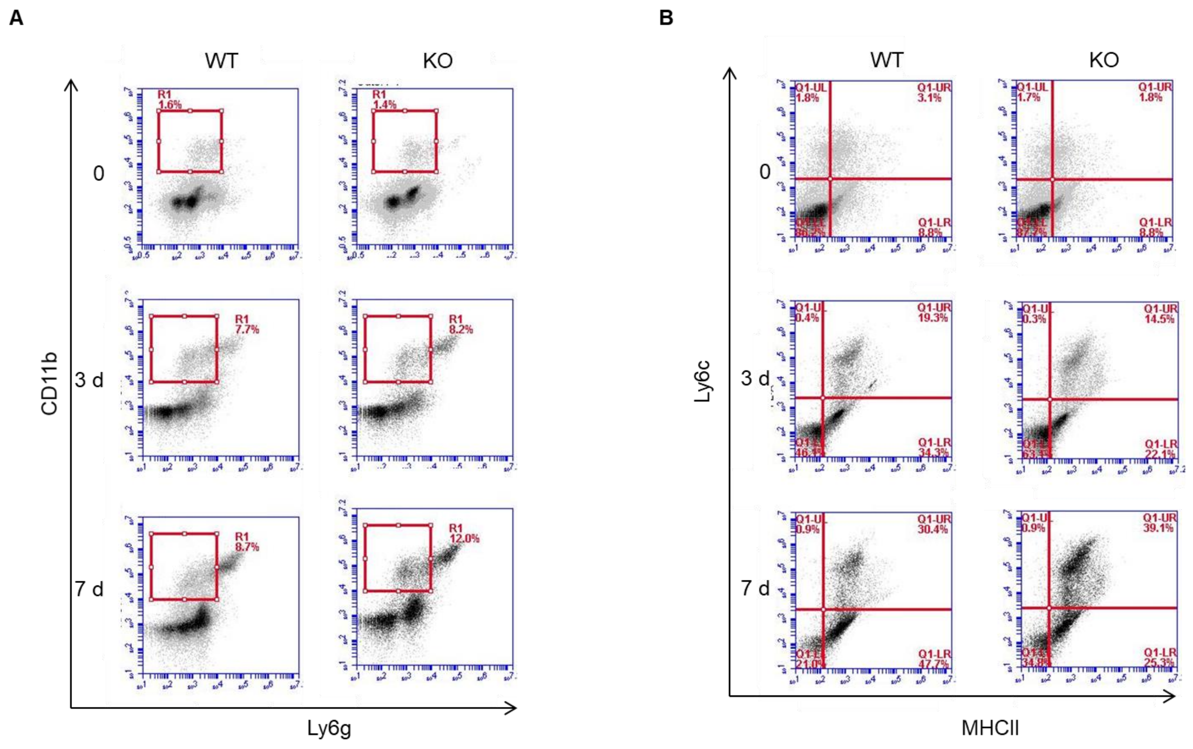

Figure S1. Representative flow cytometry plots of wound associated macrophages (WAM) analysis from skin wounds. Single-cell suspensions were prepared by enzymatic digestion and gentle dissociation of skin wounds at 0, 3, and 7 days after wounding. After excluding dead cells, the remaining cells were analyzed by flow cytometry. **(A)** WAM population was identified as F4/80^{hi}CD11b^{hi}Ly6g^{lo}. **(B)** WAM was further analyzed based on Ly6c and MHCII cell surface expression.

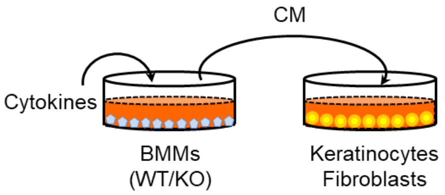

Figure S2. Graphical representation of the wound scratch assay.

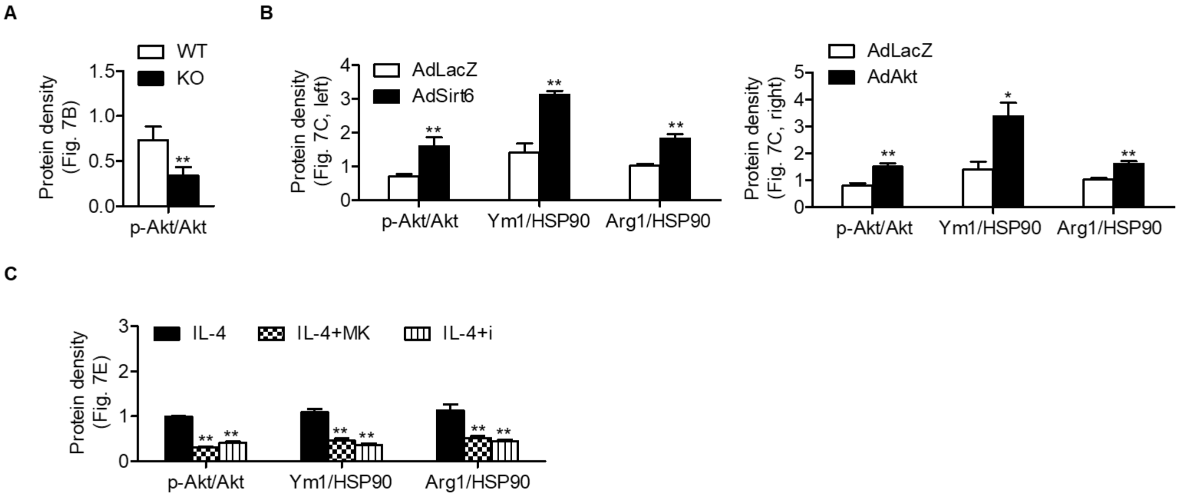

Figure S3. Densitometric analysis of Western blots. The band intensities shown in Figure 7 were quantified by densitometry (n=3). The band intensity of each protein was normalized to the intensity of Akt or HSP90 band. Values are the mean±SEM. *, p<0.05 and **, p<0.01 versus WT, AdLacZ, or IL-4.

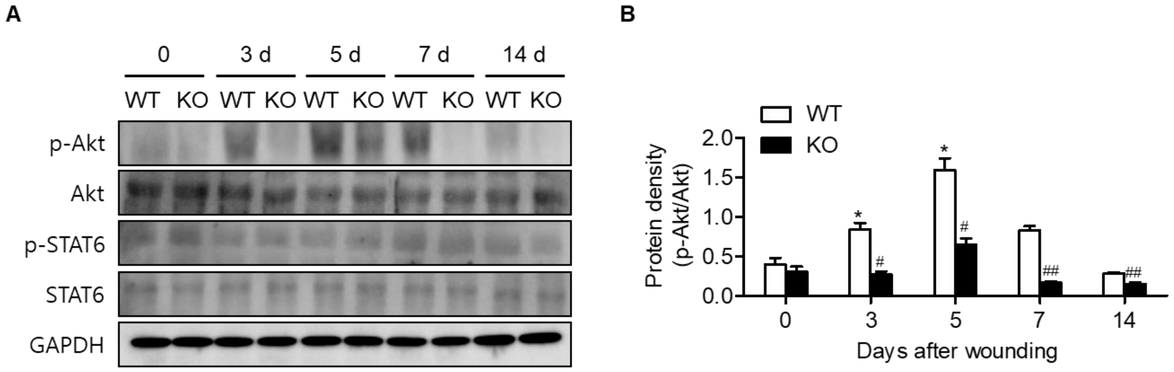

Figure S4. Decrease of Akt phosphorylation in the wound sites of mS6KO mice. (A) Skin wounds from WT or KO mice were immunoblotted with antibodies against p-Akt and p-STAT6. **(B)** The band intensities were quantified by densitometry (n=3). Values are the mean±SEM. *, p<0.05 versus Day 0; #, p<0.05 and ##, p<0.01 vs. WT.
